# Transient ischemic attack and acute ischemic stroke: evidence of altered corrected index of cardiac electrophysiological balance

**DOI:** 10.3389/fneur.2025.1688434

**Published:** 2026-01-08

**Authors:** Zhen Hu, Chan Gao, Xiaoping Yu, Ankang Yang, Lin Zhu, Yuying Deng, Shuo Zhang, Yafei Peng, Kuangrong Song, Liangqiao Zhao, Hanqin Huang, Jiajia Lu

**Affiliations:** Department of Electrocardiography Diagnosis, Wuhan No.1 Hospital, Wuhan, Hubei, China

**Keywords:** transient ischemic attack, acute ischemic stroke, index of cardiac electrophysiological balance, corrected index of cardiac electrophysiological balance, National Institutes of Health Stroke Scale

## Abstract

**Background:**

Several electrocardiogram (ECG) abnormalities have been reported in patients with acute cerebral ischemic events. The index of cardiac electrophysiological balance (iCEB) and corrected index of cardiac electrophysiological balance (iCEBc) are potential non-invasive markers for arrhythmogenesis. Therefore, Chinese patients diagnosed with transient ischemic attack (TIA) or acute ischemic stroke (AIS) were included in this case–control study to explore the potential alterations in these markers and to determine the relationships between them and the National Institutes of Health Stroke Scale (NIHSS) score at admission.

**Methods:**

We performed a hypothesis-generating, exploratory analysis on retrospective data. Consecutive patients diagnosed with TIA or AIS at the Department of Neurology, Wuhan No. 1 Hospital, from January 2021 to June 2024, were included. Patients with bundle branch block, intraventricular conduction delay, ventricular pacing, ventricular rhythm, sino-ventricular conduction, atrial fibrillation or atrial flutter, Wolff-Parkinson-White, long QT syndrome, thyroid dysfunction, serum electrolyte imbalances, acute coronary syndrome, acute heart failure, severe valvular heart disease, cardiomyopathy were excluded from the study. There are several formulas for calculating QTc. Given the computational dependency of iCEBc on QTc values, we specifically investigated the potential formula-related effects of two variants: iCEBcB (based on Bazett’s formula) and iCEBcF (based on Fridericia’s formula). ICEB, iCEBcB, and iCEBcF were compared between groups. Regression analyses demonstrated connections between these variables and the NIHSS score in patients with AIS at the time of admission.

**Results:**

A total of 382 Chinese patients were enrolled in this study. There were 52 in the control group, 96 in the TIA group, and 234 patients in the AIS group. From the control group, through the TIA group, to the AIS group, both iCEBcB and iCEBcF demonstrated a gradual upward trend. Only iCEBcB showed an independent and positive correlation with the NIHSS score at admission.

**Conclusion:**

This study identified a graded increase in iCEBcB and iCEBcB across the clinical spectrum, from controls to TIA and further to AIS patients. An elevated iCEBcB emerged as a significant independent predictor of the NIHSS score at admission. ICEBcB could provide a non-invasive means of detecting early, subclinical electrophysiological abnormalities in patients with ischemic stroke.

## Introduction

Stroke is one of the most common causes of disability and death. Ischemic stroke ranges from transient ischemic attack to severe brain damage. Acute ischemic stroke (AIS) is defined as sudden neurological dysfunction caused by focal brain ischemia, with imaging demonstrating acute infarction. Transient ischemic attack (TIA) is characterized by ischemic episodes with neurologic deficits, but no evidence of acute infarction on imaging. The TIA serves as a precursor for AIS (1). Cardiovascular morbidity and mortality after stroke are both critical determinants of long-term prognosis and a major clinical challenge (2). Therefore, early detection of cardiovascular complications is essential in clinical practice, demanding innovative approaches for stroke survivors (3).

Normal cardiac electrophysiology relies on the organized propagation of electrical impulses, producing rapid depolarization followed by slow repolarization. Such sequential activation induces myocyte action potentials, thereby ensuring an effective contraction (4). ECG is a cost-effective method for assessing myocardial depolarization and repolarization dynamics. Disturbances in these two systems result in arrhythmias. Several novel ECG parameters provide insight into cardiac electrophysiological stability in distinct clinical contexts (5–8). The reproducible and computational data affirm the vibrant research potential in this field. Integration of such markers with data-driven methods may have a chance to unlock deeper clinical insights from it. The iCEB is quantified using the ratio of the QT interval to the QRS duration (QT/QRS). The iCEBc is the corrected version that accounts for variations in the heart rate (QTc/QRS). ICEB evaluates both the excitation and recovery phases of cardiac electrical activity. Therefore, abnormal iCEB and iCEBc levels provide a broader perspective on the risk of arrhythmias (9, 10). In addition, iCEB and iCEBc have demonstrated significant prognostic value in predicting major adverse cardiac events, cardiac mortality, and all-cause death (11, 12). The existing literatures provide limited data on the values of iCEB and iCEBc in patients with ischemic stroke (13, 14). This study was motivated by two critical considerations. First, there is growing recognition of ethnic differences in fundamental electrocardiographic parameters (15). The existing literature on this association is predominantly derived from Turkish populations, and its generalizability to Chinese individuals remains unclear. Second, the choice of formula for heart rate correction of the QT interval (QTc) can influence research findings and clinical interpretation (16, 17). Therefore, we aimed to evaluate these parameters in Chinese patients with TIA and AIS using the two most prevalent formulae, Bazett’s and Fridericia’s. We also examined the potential associations between these parameters and the NIHSS score.

## Materials and methods

This case–control study was approved by the Institutional Ethics Committee of Wuhan No.1 Hospital (Ethical Application Ref: 2024-63) and conducted in accordance with the Declaration of Helsinki. The requirement for informed consent was waived by the Ethics Committee owing to the retrospective nature of this study.

### Inclusion criteria

Medical records of consecutive patients diagnosed with TIA or AIS were reviewed. All participants were hospitalized at the Department of Neurology, Wuhan No.1 Hospital, from January 2021 to June 2024. The inclusion criteria were as follows: (1) age ≥ 18 years, (2) diagnosis of TIA or AIS, and (3) 12-lead ECG on admission. The diagnoses of TIA and AIS were definitively established utilizing neuroimaging, either by computed tomography or magnetic resonance imaging. The control group in this study consists of age and gender matched inpatients who were admitted for surgical treatment of renal cysts detected during physical examinations.

### Exclusion criteria

Electrocardiographic Exclusions: evidence of bundle branch block (left or right), intraventricular conduction delay (QRS duration > 110 ms), presence of a paced ventricular rhythm, ventricular rhythm (ventricular escape rhythm, idio-ventricular rhythm, ventricular tachycardia), sino-ventricular conduction, atrial fibrillation or atrial flutter, Wolff-Parkinson-White, congenital or acquired long QT syndrome (QTc interval ≥ 500 ms). Clinical Exclusions: thyroid dysfunction (hyperthyroidism, hypothyroidism), serum electrolyte imbalances (potassium, sodium, calcium, magnesium), acute coronary syndrome, acute heart failure, severe valvular heart disease (severe stenosis or regurgitation on echocardiography), cardiomyopathy (dilated, hypertrophic, or restrictive).

### Assessment of clinical characteristics

The following variables were collected from the participants: sex, age, systolic blood pressure (SBP), diastolic blood pressure (DBP), and standard blood tests including potassium, sodium, calcium, magnesium, glucose, triglyceride (TG), total cholesterol (TC), high-density lipoprotein (HDL) cholesterol, low-density lipoprotein (LDL) cholesterol, and the NIHSS score at the time of admission.

### Assessment of ECG

Standard 12-lead ECGs acquired during admission were systematically analyzed. The recordings were performed at 25 mm/s and 10 mm/mv. Most ECGs were obtained on the day of admission, with the remainder completed on the day before admission or on the day after admission. These parameters were obtained by using an automated ECG analysis system (Imedway, EKGStudio version 4.9.1). Heart rate was defined as beats per minute. QRS duration was established from the beginning to the end of the QRS wave. The QT interval was measured from the onset of the QRS wave to the end of the T wave. QRS duration and QT interval were measured by superposition of the 12 ECG leads. QTc interval was the heart-rate-corrected version of the QT interval. ICEB was calculated as the ratio of the QT interval to the QRS duration (iCEB = QT/QRS). iCEBc represents a rate-corrected iCEB value. It was calculated as the ratio of the QTc interval to the QRS duration (iCEBc = QTc/QRS). To assess the variability of the correction formula, we compared the two most widely used methods: Bazett’s (QTcB = QT/[√ RR]) and Fridericia’s (QTcF = QT interval/(RR interval)^1/3^). We also derived two variants of iCEBc: iCEBcB (QTcB/QRS) and iCEBcF (QTcF/QRS) to evaluate formula-dependent effects. Four independent experts reevaluated the measurements, and corrections were made where necessary.

### Statistical analyses

Statistical analyses were performed using IBM SPSS Statistics version 22 and R software Version 4.1.1 with RStudio. Categorical variables were described as frequencies and percentages. Between-group comparisons of categorical variables were performed using the chi-square test. Continuous variables were assessed for normality (Shapiro–Wilk test) and homogeneity of variance (Levene’s test). Normal data were presented as mean ± standard deviation. Non-normally distributed data were reported as median with interquartile range (Q1–Q3). A parametric test was performed using analysis of variance (ANOVA). A non-parametric test was performed using the Kruskal–Wallis test. Dunn’s test with Bonferroni correction was used for post-hoc pairwise comparisons after the Kruskal–Wallis test. Linear regression analysis was used to identify the significant indicators. Variables with *p* < 0.05 in univariate analysis were included in the multivariate model. For parameters (HR, QTc, and iCEBc) with potential multicollinearity issues, we present three alternative models for clarity and robustness. Statistical significance was set at *p* < 0.05.

## Results

### Clinical characteristics and laboratory parameters

Table 1 presents the baseline clinical characteristics and laboratory parameters of patients. In total, 382 patients were enrolled in this study: 52 in the control group, 96 in the TIA group, and 234 in the AIS group. Hypertension was most prevalent in the AIS group (176/234, *p* = 0.000), whereas hyperlipidemia was most frequently observed in the TIA group (42/96, *p* = 0.000). Regarding cardiac conditions, the control group exhibited a higher proportion of mild valvular disease (5/52, *p* = 0.000). Patients in the AIS group had higher SBP [142 (130, 160), *p* = 0.000] and glucose level [6.8(5.5, 8.6), *p* = 0.000]. Additionally, the other variables did not differ significantly among the three groups (*p* ≥ 0.05).

**Table 1:** Clinical characteristics and laboratory parameters among groups.

Variables	Control patients (*n* = 52)	TIA patients (*n* = 96)	AIS patients (*n* = 234)	*p* value
Gender (male), *n* %	35/52	57/96	149/234	0.606
Age (year)	64 (60, 68)	66 (60, 70)	65 (59, 72)	0.410
hypertension	24/52	49/96	176/234	0.000
diabetes mellitus	8/52	22/96	71/234	0.057
hyperlipidemia	5/52	42/96	82/234	0.000
Coronary heart disease	1/52	13/96	28/234	0.073
Cardiac dysfunction	0/52	0/96	7/234	0.105
Arrhythmia	2/52	14/96	23/234	0.114
Valvular heart disease	5/52	5/96	1/234	0.000
SBP (mmHg)	135 (124, 143)	137 (124, 148)	142 (130, 160)	0.000
DBP (mmHg)	82 (75, 87)	80 (77, 90)	82 (77, 90)	0.499
Potassium (mmol/L)	3.9 (3.7, 4.0)	3.9 (3.7, 4.2)	3.9 (3.7, 4.1)	0.600
Sodium (mmol/L)	140.9 (139.2, 142.6)	140.8 (139.7, 141.8)	141.0 (139.4, 142.6)	0.589
Calcium (mmol/L)	2.27 (2.20, 2.33)	2.26 (2.19, 2.35)	2.25 (2.19, 2.33)	0.554
Magnesium (mmol/L)	0.85 (0.83, 0.89)	0.87 (0.80, 0.91)	0.85 (0.80, 0.90)	0.469
Glucose (mmol/L)	5.0 (4.5, 5.5)	5.5 (4.8, 6.7)	6.8 (5.5, 8.6)	0.000
TG (mmol/L)	1.36 (1.07, 1.76)	1.26 (0.99, 1.78)	1.35 (1.04, 1.90)	0.538
TC (mmol/L)	4.64 (4.08, 5.12)	4.35 (3.56, 5.33)	4.38 (3.60, 5.19)	0.468
HDL cholesterol (mmol/L)	1.07 (0.93, 1.24)	1.09 (0.95, 1.32)	1.10 (0.91, 1.28)	0.921
LDL cholesterol (mmol/L)	3.13 (2.74, 3.57)	2.76 (2.15, 3.46)	2.83 (2.15, 3.42)	0.051

TIA, transient ischemic attack; AIS, acute ischemic stroke; SBP, systolic blood pressure; DBP, diastolic blood pressure; TG, triglyceride; TC, total cholesterol; HDL, high-density lipoprotein; LDL, low-density lipoprotein.

### ECG indicators

Table 2 summarizes the ECG characteristics of the study population.

**Table 2:** ECG indicators among groups.

Variables	Control patients (*n* = 52)	TIA patients (*n* = 96)	AIS patients (*n* = 234)	*p* value
HR (bpm)	70 (63, 78)	70 (62, 78)	73 (64, 82)	0.133
QRS (ms)	90 (84, 95)	90 (84, 95)	88 (83, 95)	0.646
QT interval (ms)	386 (369, 407)	401 (378, 416)	394 (370, 421)	0.195
QTcB interval (ms)	420 ± 21	429 ± 25	434 ± 25	0.001
QTcF interval (ms)	409 ± 19	418 ± 22	420 ± 23	0.009
iCEB	4.36 ± 0.47	4.47 ± 0.52	4.48 ± 0.55	0.327
iCEBcB	4.69 (4.48, 5.03)	4.84 (4.51, 5.16)	4.93 (4.55, 5.33)	0.027
iCEBcF	4.59 ± 0.41	4.7 ± 0.48	4.77 ± 0.51	0.037

TIA, transient ischemic attack; AIS, acute ischemic stroke; HR, heart rate; QTcB interval, QTc interval based on Bazett’s formula; QTcF interval, QTc interval based on Fridericia’s formula; iCEB, index of cardiac electrophysiological balance; iCEBcB, corrected index of cardio-electrophysiological balance based on Bazett’s formula; iCEBcF, corrected index of cardio-electrophysiological balance based on Fridericia’s formula.

**Figure 1:**
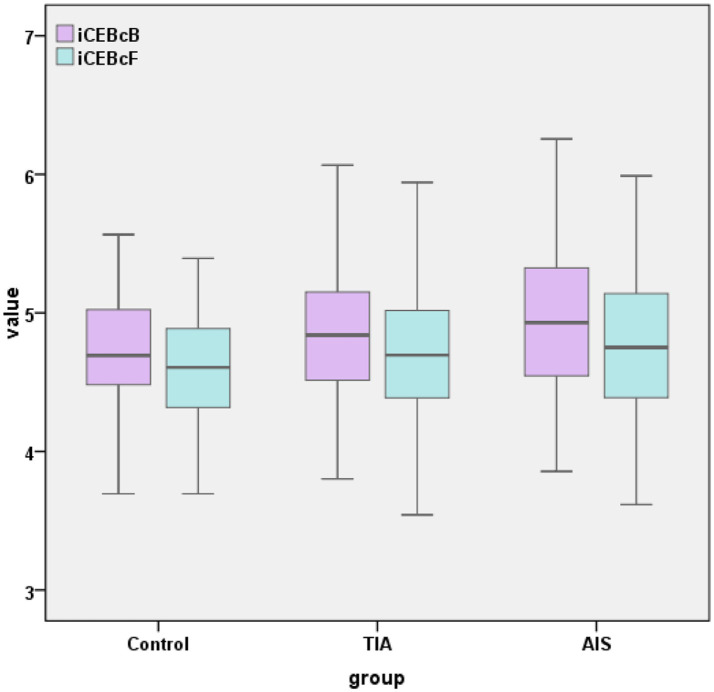
A boxplot for comparison of iCEBcB and iCEBcF among groups. TIA, transient ischemic attack; AIS, acute ischemic stroke; iCEBcB, corrected index of cardio-electrophysiological balance based on Bazett’s formula; iCEBcF, corrected index of cardio-electrophysiological balance based on Fridericia’s formula.

The AIS group demonstrated significantly higher values in QTcB (434 ± 25, *p* = 0.001), QTcF (420 ± 23, *p* = 0.009), iCEBcB [4.93(4.55, 5.33), *p* = 0.027], and iCEBcF (4.77 ± 0.51, *p* = 0.037) with statistical significance. Other measured indicators showed no statistically significant intergroup differences. As illustrated in Figure 1, the box plots depict the distribution of both iCEBcB and iCEBcF values across the groups. Visually, both iCEBc showed a similar trend of elevation from the control group to the TIA group, and further to the AIS group.

### Pairwise comparisons of iCEBcB across groups

Table 3 shows the post-hoc analyses with Bonferroni correction of iCEBcB across groups. The result revealed the main statistical difference in iCEBcB values between AIS vs. control groups (Adjusted *p*-value = 0.031).

**Table 3:** Pairwise comparisons of iCEBcB between groups: effect sizes and post-hoc test results.

Variables	Comparison	Effect size (median difference)	95% CI	*Z* statistic	Unadjusted *p*-value	Adjusted *p*-value
iCEBcB	TIA vs. Control	0.15	(0.00 to 0.30)	−1.307	0.191	0.574
	AIS vs. Control	0.24	(0.12 to 0.37)	2.560	0.010	0.031
	AIS vs. TIA	0.09	(−0.07 to 0.23)	1.381	0.167	0.501

TIA, transient ischemic attack; AIS, acute ischemic stroke; iCEBcB, corrected index of cardio-electrophysiological balance based on Bazett’s formula.

### Associations between NIHSS and other variables

**Figure 2:**
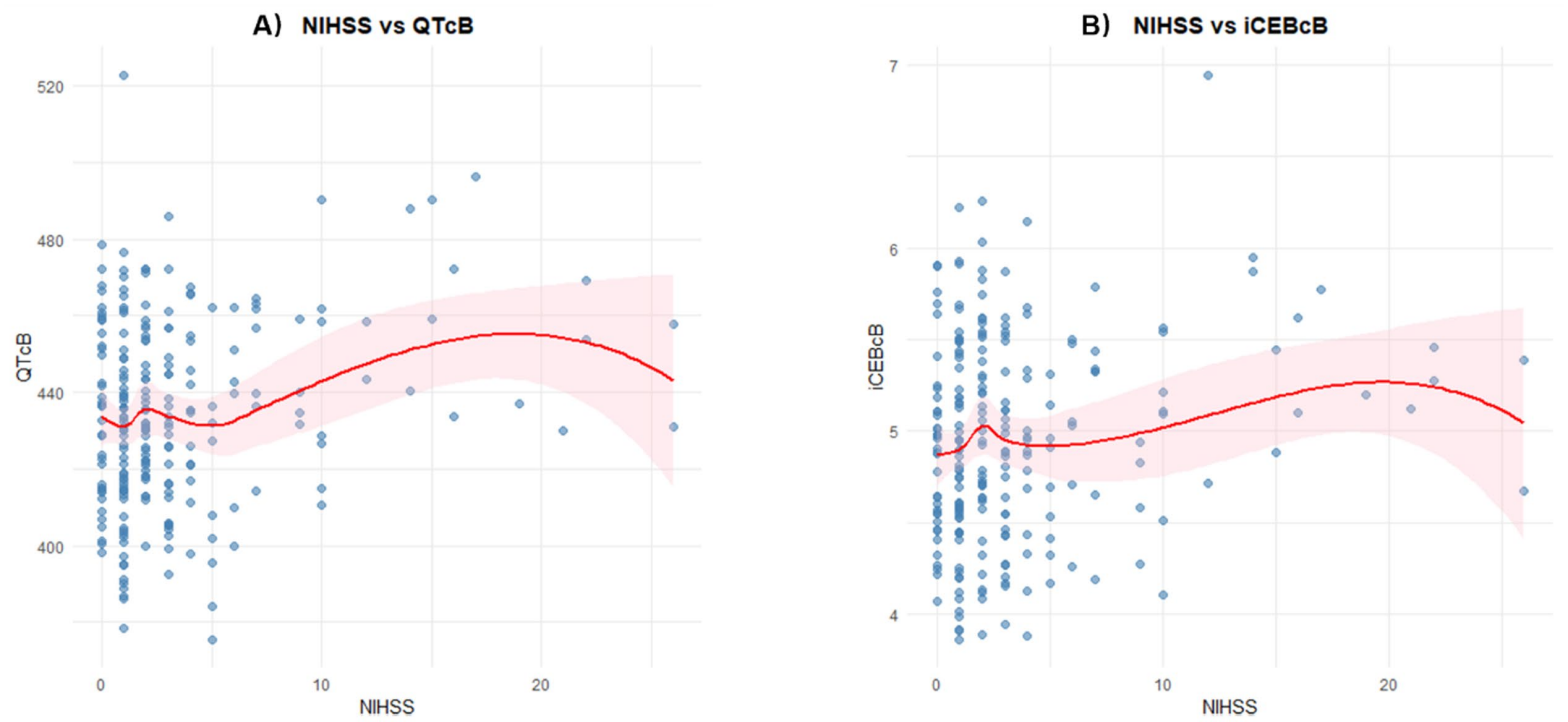
Relationships between NIHSS scores and electrocardiographic parameters with LOESS smoothing: **(A)** QTcB and **(B)** iCEBcB. NIHSS, National Institutes of Health Stroke Scale; QTcB interval, QTc interval based on Bazett’s formula; iCEBcB, corrected index of cardio-electrophysiological balance based on Bazett’s formula.

Table 4 shows the associations between NIHSS score and other variables in patients with AIS. Univariate regression analysis showed that age (*β* = 0.056, *p* = 0.035), SBP (*β* = 0.033, *p* = 0.013), calcium (*β* = −11.222, *p* = 0.000), HR (*β* = 0.091, *p* = 0.000), QTcB interval (*β* = 0.041, *p* = 0.001), and iCEBcB (*β* = 1.464, *p* = 0.007) were significantly associated with the NIHSS score. The LOESS curves in Figure 2 illustrate the relationship between QTcB, iCEBcB, and NIHSS scores. The above-mentioned parameters were further incorporated into the multivariate regression analysis. To address potential multicollinearity among key electrophysiological parameters (HR, QTcB, and iCEBcB), we constructed three distinct regression models. The association for iCEBcB remained statistically significant (*β* = 1.342, *p* = 0.019) in Model C, independent of HR and QTcB, which were excluded from this model.

**Table 4:** Regression analysis of parameters significantly correlated with NIHSS in patients with AIS.

Variables	NIHSS in patients with AIS (*n* = 234)							
	Univariable analysis		Multivariable analysis					
			Model A (with HR)		Model B (with QTcB)		Model C (with iCEBcB)	
	*β*	*p*	*β*	*p*	*β*	*p*	*β*	*p*
Age (y)	0.056	0.035	0.042	0.115	0.030	0.269	0.025	0.367
SBP (mmHg)	0.033	0.013	0.030	0.022	0.033	0.016	0.036	0.009
Calcium (mmol/L)	−11.222	0.000	−9.662	0.001	−9.929	0.001	−10.104	0.001
HR (bpm)	0.091	0.000	0.085	0.000	/		/	
QTcB interval (ms)	0.041	0.001	/		0.036	0.004	/	
iCEBcB	1.464	0.007	/		/		1.342	0.019
Max VIF	/		1.030		1.047		1.090	

Model A (containing HR but not QTcB and iCEBcB), Model B (containing QTcB but not HR and iCEBcB), and Model C (containing iCEBcB but not HR and QTcB).

NIHSS, National Institutes of Health Stroke Scale; AIS, acute ischemic stroke; SBP, systolic blood pressure; HR, heart rate; QTcB interval, QTc interval based on Bazett’s formula; iCEBcB, corrected index of cardio-electrophysiological balance based on Bazett’s formula.

### Associations between the iCEBcB and other variables

Table 5 summarizes the association between iCEBcB level and other variables in patients with AIS. ICEBcB was influenced by age (*β* = 0.012, *p* = 0.000), DBP (*β* = −0.009, *p* = 0.004), and NIHSS (*β* = 0.021, *p* = 0.007). Only age (*β* = 0.009, *p* = 0.004) and NIHSS score (*β* = 0.017, *p* = 0.030) were confirmed in multivariate regression analysis.

**Table 5:** Regression analysis of parameters significantly correlated with iCEBcB in patients with AIS.

Variables	iCEBcB in patients with AIS (*n* = 234)			
	Univariable analysis		Multivariable analysis	
	*β*	*p*	*β*	*p*
Age (year)	0.012	0.000	0.009	0.004
DBP (mmHg)	−0.009	0.004	−0.005	0.077
NIHSS	0.021	0.007	0.017	0.030

iCEBcB, corrected index of cardio-electrophysiological balance based on Bazett’s formula; AIS, acute ischemic stroke; DBP, diastolic blood pressure; NIHSS, National Institutes of Health Stroke Scale.

## Discussion

Ischemic stroke is a devastating disease. Arrhythmias following AIS can induce hemodynamic instability and lead to worse clinical outcomes. Markers that identify high-risk individuals are valuable for early clinical intervention. The cardiac excitation wavelength *λ* was calculated as the product of the effective refractory period (ERP) and conduction velocity (CV). It has been demonstrated to be the best predictor of arrhythmic tendency. One study hypothesized that QT interval changes correlate proportionally with ERP variations and that QRS duration changes reflect CV modifications. Thus, iCEB (the QT/QRS ratio) was proposed as a non-invasive method analogous to the traditional λ (ERP × CV) (18). Multiple studies have shown that this index serves as a marker for arrhythmia risk across different neurological disorders (13, 14, 19, 20). This study assessed the association between ischemic stroke and ECG parameters, including iCEB, iCEBcB, and iCEBcF.

Progressive iCEBc prolongation was observed across the groups in the study, with the lowest values in the control group, intermediate values in the TIA group, and the highest values in the AIS group. In addition, the NIHSS is a simple and useful scale for clinical evaluation of patients with AIS. A higher NIHSS score indicates more severe stroke impairment. An elevated iCEBcB emerged as a significant independent predictor of the NIHSS score in the regression model in our study. These results align with previous studies (13, 14). Based on the above evidence, we hypothesized that patients with more severe cerebral ischemia would exhibit a higher susceptibility to arrhythmias, leading to a significant increase in iCEBc. From another perspective, this gradient strengthens the biological association between cerebral ischemic burden and disturbance of cardiac depolarization with repolarization. It might help in risk stratification within the spectrum of cerebrovascular events. What’s more, the clinical definitions of TIA (transient symptoms) and AIS (permanent injury) inherently reflect a difference in the extent and duration of cerebral ischemia. Our finding that iCEBc values occupy an intermediate position in TIA patients provides evidence that this cardiac phenomenon is sensitive to the gradations of brain injury, rather than being an all-or-nothing response.

The iCEBc is calculated from QTc, and different QTc formulae possess distinct heart-rate correction properties and clinical contexts (21–23). We analyzed the variability of Bazett’s and Fridericia’s correction formula via QTcB, QTcF, iCEBcB, and iCEBcF. While both iCEBcB and iCEBcF showed group differences, only iCEBcB emerged as a potential discriminative marker in the multivariable analysis of NIHSS. A previous study identified Bazett’s formula as the best for cardiac mortality prediction, despite demonstrating the poorest performance when assessed by mathematical methods alone (23). In our study, iCEBcB was uniquely capturing a maladaptive, brain-heart interaction specific to the acute phase of cerebral ischemia, a signal that might be ‘over-smoothed’ by Fridericia’s formulae. Given that Bazett’s formula remains the most widely used and clinically recognized correction formula in acute stroke, we recommend it as the primary choice in future analysis.

As an authoritative and widely used electrocardiographic indicator, the QT interval and QTc interval hold indispensable significance in clinical diagnosis. Evidence from end-stage renal disease patients demonstrates a significant post-dialysis increase in iCEBc, contrasting with the stability of the QTc interval (24). Another study showed that iCEBc changed before QTc did in drug-related arrhythmias, suggesting that iCEBc is more sensitive than the QTc (25). In this exploratory study, we confirmed the established association between QTc and ischemic stroke disease, thereby validating the general concept of brain-heart interaction in our study. We demonstrated that the novel index iCEBc also exhibits a significant and independent association with both disease status and severity. While iCEBc did not outperform QTc in discriminatory power in this initial investigation, its key value may lie in complementarity. In summary, the clinical application of iCEBc deserves further attention. Future research should focus on identifying the specific scenarios in which iCEBc provides a distinct aspect of stroke-induced cardiac vulnerability that is not fully reflected by QTc alone.

### Study limitations

Our study had several limitations. First, the study was conducted at a single center with a limited number of patients. Second, owing to the retrospective nature of the study, we were unable to follow up on the terms of malignant ventricular arrhythmias or sudden cardiac death. Third, the absence of pharmacotherapy records might represent a potential source of bias. Fourth, the strict exclusion of patients with major cardiovascular comorbidities enhanced internal validity but excluded many severe cases (including high-risk cardiac patients) that could interfere with results. This introduces potential sample composition imbalance and limits generalizability to real-world stroke populations. Prospective multicenter studies with larger samples are needed to address these limitations. Specifically, future research should aim to characterize the temporal profile of the biomarker, alongside conducting subgroup analysis of patients with major cardiovascular comorbidities (including medication information) and long-term monitoring assessments, to provide a more comprehensive understanding.

## Conclusion

Our study provides evidence of a trend of gradual increase in iCEBcB and iCEBcF levels in the control, TIA, and AIS groups. Moreover, the iCEBcB was identified as an independent predictor of NIHSS score at admission. In conclusion, iCEBcB may serve as a novel marker for identifying patients at higher risk of arrhythmia.

## Data Availability

The raw data supporting the conclusions of this article will be made available by the authors, without undue reservation.
